# Continuous dynamic identification of key genes and molecular signaling pathways of periosteum in guided bone self-generation in swine model

**DOI:** 10.1186/s13018-023-03524-y

**Published:** 2023-01-18

**Authors:** Bao-Fu Yu, Zi Wang, Xiao-Xue Chen, Qi Zeng, Chuan-Chang Dai, Jiao Wei

**Affiliations:** 1 Department of Plastic and Reconstructive Surgery, Shanghai Ninth People’s Hospital Affiliated to Shanghai Jiaotong University School of Medicine, No. 639 Zhi Zao Ju Road, Shanghai, 200011 China; 2 Department of Plastic Surgery, Jiangxi Province People’s Hospital, Nanchang, China

**Keywords:** Guided bone self-generation, Periosteum, Transcriptome, Signal pathway, Gene expression

## Abstract

**Background:**

Guided bone self-generation with periosteum-preserved has successfully regenerated mandibular, temporomandibular and interphalangeal joint. The aim of this study was to investigate the dynamic changes of gene expression of periosteum which was involved in the guided bone self-generation.

**Methods:**

Rib defects of critical size were created in mature swine with periosteum-preserved. The periosteum was sutured into a sealed sheath that closed the bone defect. The periosteum of trauma and control sites were harvested at postoperative 9 time points, and total RNA was extracted. Microarray analysis was conducted to identify the differences in the transcriptome of different time points between two groups.

**Results:**

The differentially expressed genes (DEGs) between control and trauma group were different at postoperative different time points. The dynamic changes of the number of DEGs fluctuated a lot. There were 3 volatility peaks, and we chose 3 time points of DEG number peak (1 week, 5 weeks and 6 months) to study the functions of DEGs. Oxidoreductase activity, oxidation–reduction process and mitochondrion are the most enriched terms of Go analysis. The major signaling pathways of DEGs enrichment include oxidative phosphorylation, PI3K-Akt signaling pathway, osteoclast differentiation pathway and Wnt signaling.

**Conclusions:**

The oxidoreductase reaction was activated during this bone regeneration process. The oxidative phosphorylation, PI3K-Akt signaling pathway, osteoclast differentiation pathway and Wnt signaling may play important roles in the guided bone self-generation with periosteum-preserved. This study can provide a reference for how to improve the application of this concept of bone regeneration.

**Supplementary Information:**

The online version contains supplementary material available at 10.1186/s13018-023-03524-y.

## Introduction

Periosteum, a highly vascularized connective tissue, plays a key role in the growth, development and regeneration of bone [1]. It covers the external surface of bones except for the sites of articulation surface and muscle attachment. The inner cambium layer of periosteum contains skeletal stem cell population [2]. The skeletal stem cell population roles importantly in the fracture healing and bone development, which could differentiate into chondrogenic and osteogenic lineages [3, 4].

Bone formations include intramembranous bone formation and endochondral bone formation [5]. During the process, the periosteum lays down intramembranous woven bone. The tissue damage closer to the fracture site could block the blood supply and result in the formation of cartilaginous masses [6]. Then the endochondral bone formation was observed. The chondrocytes were reported to derive predominantly from the periosteum. Therefore, periosteum is key factor in the two kinds of bone formation.

With high regenerative capacity, periosteum has high potential of the utilization for bone regeneration [7]. In clinical studies, it has been reported that spontaneous bone regeneration could be observed to re-establish the bony continuity for bone tumor segmentectomy with periosteum preservation [8, 9]. This phenomenon provides enlightening resolution for endogenous bone tissue engineering for bone defects. We have created animal models of bone defects to study the periosteum and bone regeneration, and reconstructed new bone tissues which were histologically, anatomically and functionally similar to normal bone tissue [10–12]. The guided production of autologous bone without exogenous cells, scaffolds and growth factors is a novel concept deserving exploring in further clinical application like self-generation joint and cartilage. Therefore, a better understanding of molecular mechanisms is important for accelerating the bone regeneration process.

In this study, we used microarray to investigate the dynamic changes of gene expression of the periosteum involved in the bone regeneration for bone defect at different time points. The study may help us to have a better understanding of the molecular regulation mechanisms that periosteum is involved in during the bone regeneration of periosteum-preserved bone defect.

## Materials and methods

This study was approved by the Animal Research Ethics Committee of the authors’ institution and carried out in accordance with the approved guidelines (SH9H-2019-A645-1).

### Subjects and surgical procedure

**Fig. 1:**
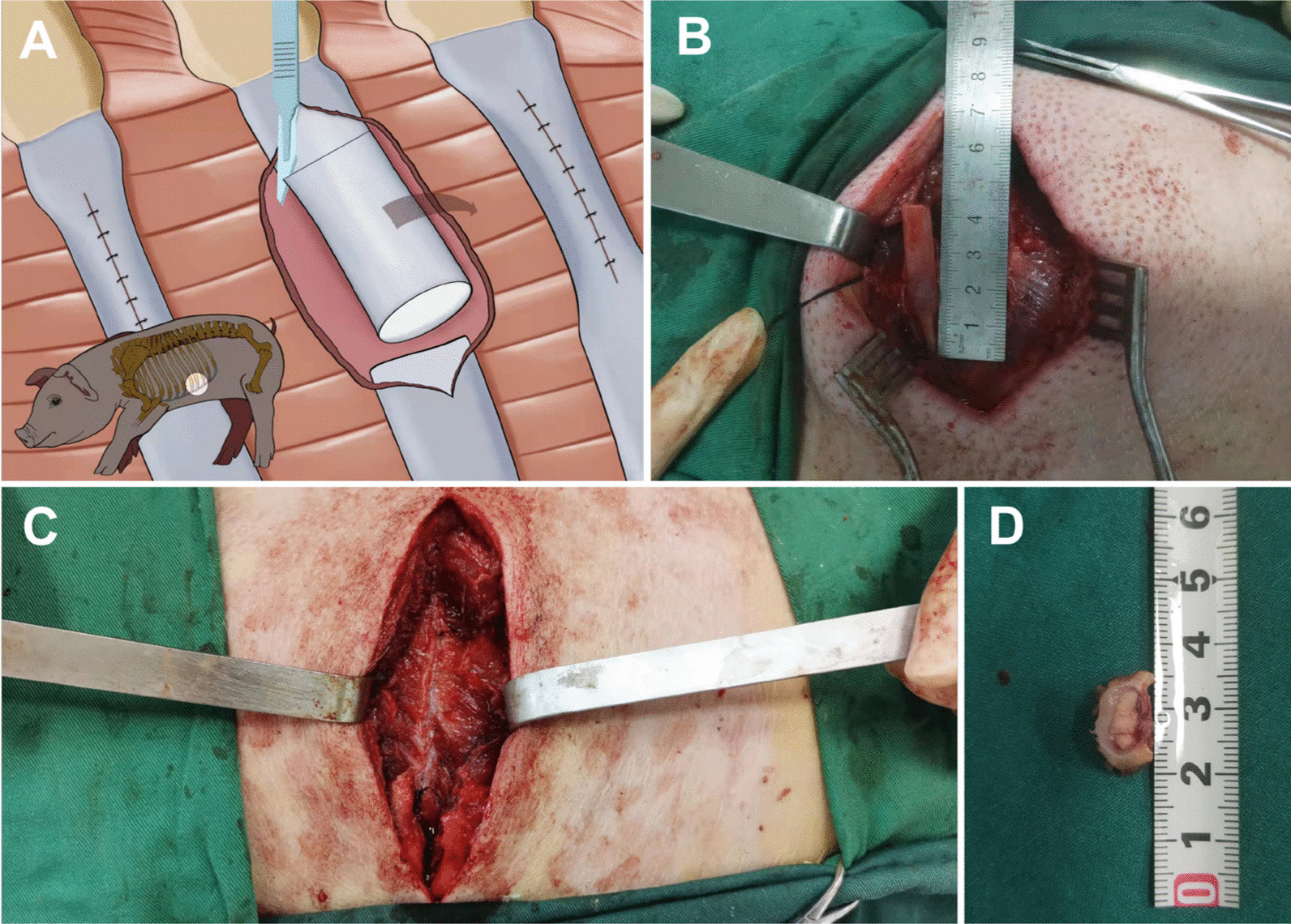
Establishment of the animal models. **A** Schematic diagram of this procedure. **B** A segment of rib was removed, which resulted in a 3 cm defect of rib. **C** The incised periosteum was sutured, creating a closed space. **D** The periostea were harvested after the swine were killed

Thirty-six healthy skeletally mature female swine were used in this study. The age was ranging from 4 to 6 months. Under general anesthesia, the left side of ribs was chosen as experimental group and the right side as control group. Figure 1a shows the schematic diagram of this procedure. At the surface of the fourth rib, the chondro-osseous transition point was touched and an incision beginning from the transition point and extending for 3 cm toward the spine was created. The third, fourth and fifth ribs were exposed. The periosteum was incised at the middle of the ribs, and a segment of rib was removed, resulting in a 3 cm defect (Fig. 1b). The periosteum was sutured with 6-0 PDS sutures, creating a closed space and forming a sealed sheath (Fig. 1c). Then the wound was closed.

### Next-generation sequencing (NGS)

On postoperative 1 day, 3 days, 1 week, 2 weeks, 1 month, 5 weeks, 3 months, 6 months and 7 months, the swine were killed, and the periostea were harvested (Fig. 1d). The periostea were placed into cryotube for RNA-seq. The periostea and formed bone were stained to observe the osteogenesis. The process includes total RNA extraction, quality detection, mRNA purification, mRNA fragmentation, cDNA synthesis, PCR enrichment of library fragments, library quality inspection and on-machine sequencing.

The raw data were filtered, and the filtered high-quality sequence was compared to the reference genome. The expression level of each gene was calculated. Further analysis of expression differences, enrichment analysis and cluster analysis were performed. We perform FPKM density distribution analysis, saturation analysis, sample correlation test and principal components analysis (PCA) analysis. DESeq software package in the R language was used to analyze the PCA principal components based on the expression level.

The R language ggplots2 software package was used to draw volcano maps of DEGs. R language Pheatmap software package was used to perform two-way clustering analysis on the union of different genes and samples of all comparison groups, clustering according to the expression level of the same gene.

TopGO was used for GO enrichment analysis. GO classification was carried out according to molecular function (MF), biological process (BP) and cell component (CC). The enrichment degree was measured by Rich factor and FDR value. The top 20 GO term entries with the smallest FDR value were displayed. According to the KEGG enrichment analysis results of DEGs, the degree of enrichment was measured by Rich factor and FDR value. The top 20 KEGG pathways with the smallest FDR value were also displayed.

### Statistical analysis

Data are expressed as the mean ± S.D. Fold change and P value were calculated with *t*-test to identify DEGs. The threshold set for differentially expressed genes was the |log2FoldChange|> 1 and the *P*-value < 0.05. Functional analysis of these DEGs was conducted with associated database mentioned in the “Materials and methods” section.

## Results

All the surgeries were completed successfully. The RNA quality inspection results suggested the RNA quality of all samples was good. Therefore, the RNA samples were reliable for further transcriptomic analysis.

**Fig. 2:**
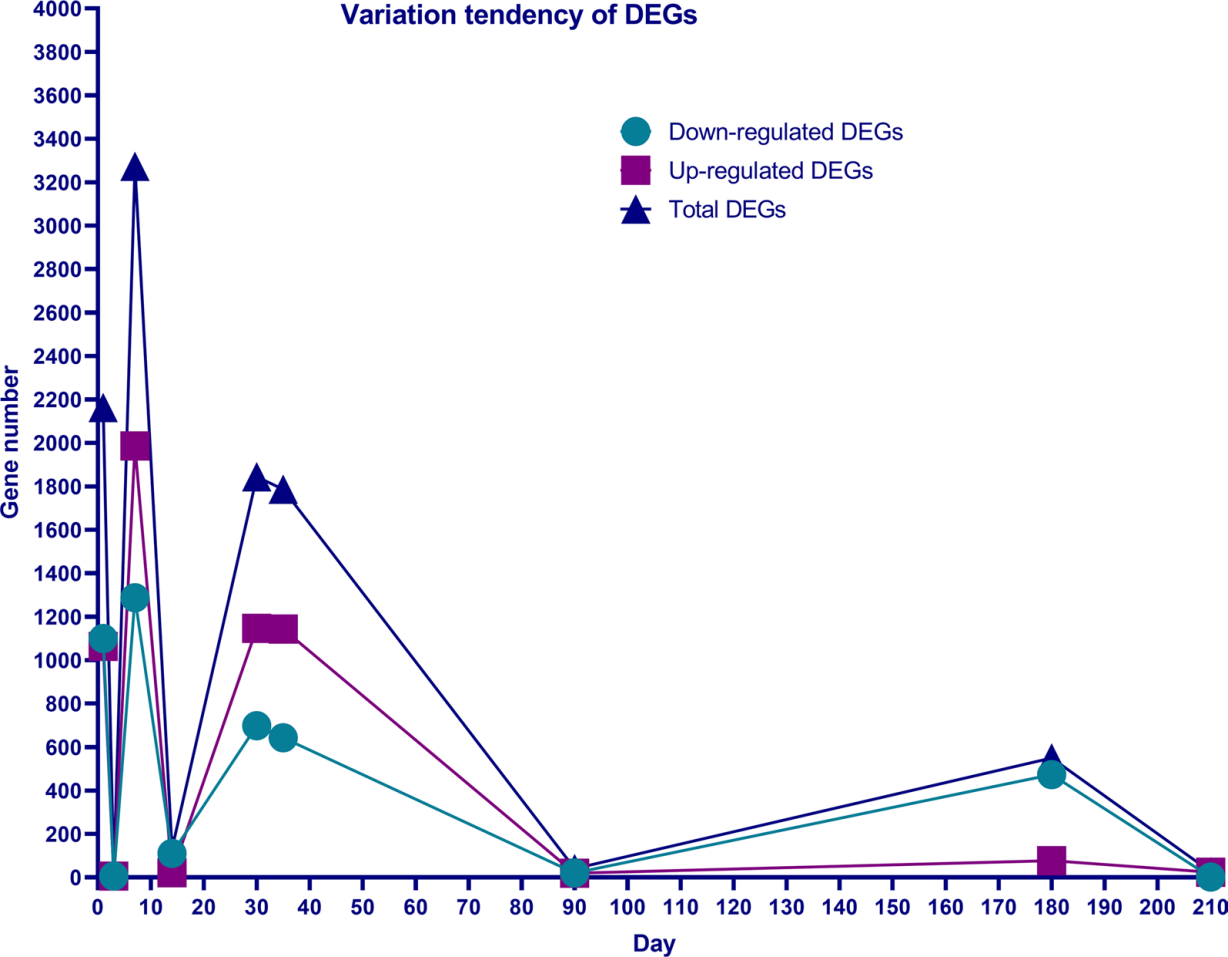
Dynamic changes of the number of DEGs, which shows that the number of up-regulated and down-regulated genes fluctuated a lot. There were 3 volatility peaks, which were similar with 3 stages of fracture healing

In Additional file 1: Fig. S1, the volcano maps show the DEGs between two groups were different at each time points. The dynamic changes of the number of DEGs are shown in Fig. 2, in which we can intuitively see that the number of up-regulated and down-regulated genes fluctuated a lot. There were 3 volatility peaks, and we chose 3 time points of the volatility peaks (1 week, 5 weeks and 6 months) to study the functions of DEGs.

Cluster analysis was used to determine the expression patterns of differentially expressed genes under different experimental conditions. Genes with high expression correlation between samples are grouped into a class; usually these genes have actual correlation in some biological process, or a metabolic or signaling pathway. Therefore, through expression clustering, we can find the unknown biological relationship between genes. The results of cluster analysis are shown in Additional file 2: Fig. S2 and show biological connections between genes. Genes are represented horizontally, with each column representing one sample, with red representing high-expressed genes and green representing low-expressed genes. From the figure, we can see that the expression levels of these genes in different samples and the expression patterns of different genes in the same sample are clustered, indicating that there are actual links in the biological processes of these genes, or in a certain metabolic or signaling pathway.

**Fig. 3:**
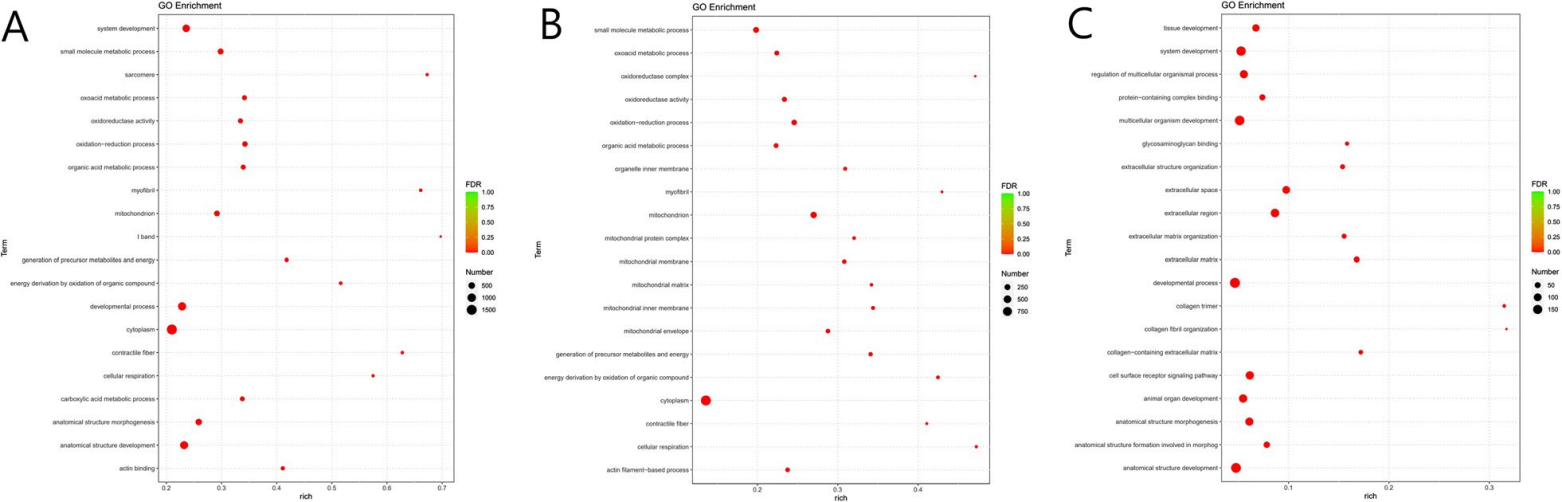
Top 20 GO term entries with the smallest FDR value. **A** Top 10 GO term entries of postoperative 1 week. **B** Top 10 GO term entries of postoperative 5 weeks. **C** Top 10 GO term entries of postoperative 6 months

The top 10 GO term entries classified into MF, BP and CC are shown in Additional file 3: Fig. S3. At postoperative 1 week, the most top terms of three categories were cytoplasm, oxidoreductase activity and oxidation–reduction process (Additional file 3: Fig. S3a). At postoperative 5 weeks, the most top terms were mitochondrion, oxidoreductase activity and oxidation–reduction process (Additional file 3: Fig. S3b). At postoperative 6 months, the most top terms were extracellular region, glycosaminoglycan binding and extracellular matrix organization (Additional file 3: Fig. S3c). The top 20 GO term entries with the smallest FDR value are shown in Fig. 3. The most significant enrichment of the top 3 GO terms according to FDR value at postoperative 1 week include oxidation–reduction process, cytoplasm and myofibril (Table 1). The most significant enrichment of the top 3 GO term entries according to FDR value at postoperative 5 weeks include mitochondrion, cytoplasm and oxidation–reduction process (Table 2). The most significant enrichment of the top 3 GO term entries according to FDR value at postoperative 6 months include extracellular region, extracellular space and extracellular matrix (Table 3).

**Table 1:** Most significant enrichment of the top 20 GO term entries according to FDR value at postoperative 1 week

Category	GO.ID	Term	Up	Down	DEG	Total	*P* value	FDR
BP	GO:0055114	Oxidation–reduction process	235	52	287	838	1.95069E−34	2.38901E−30
CC	GO:0005737	Cytoplasm	975	554	1529	7284	1.95363E−33	1.1963E−29
CC	GO:0030016	Myofibril	73	7	80	121	3.20043E−33	1.30652E−29
CC	GO:0043292	Contractile fiber	74	7	81	129	3.09568E−31	9.4782E−28
CC	GO:0030017	Sarcomere	66	6	72	107	8.92585E−31	2.1863E−27
BP	GO:0044281	Small molecule metabolic process	280	87	367	1230	6.62519E−30	1.35231E−26
BP	GO:0015980	Energy derivation by oxidation of organic compounds	90	6	96	186	6.57423E−27	1.15021E−23
BP	GO:0048856	Anatomical structure development	482	375	857	3691	1.54117E−26	2.35934E−23
BP	GO:0006082	Organic acid metabolic process	174	51	225	663	2.18282E-26	2.85419E-23
BP	GO:0043436	Oxoacid metabolic process	172	49	221	647	2.33052E−26	2.85419E−23
MF	GO:0016491	Oxidoreductase activity	182	47	229	685	4.10707E−26	4.57267E−23
BP	GO:0032502	Developmental process	511	400	911	3988	5.37969E−26	5.49043E−23
CC	GO:0005739	Mitochondrion	287	37	324	1111	2.43081E−25	2.29001E−22
MF	GO:0003779	Actin binding	81	53	134	326	8.40912E−25	7.35617E−22
BP	GO:0006091	Generation of precursor metabolites and energy	123	7	130	311	1.0409E−24	8.49862E−22
BP	GO:0045333	Cellular respiration	69	4	73	127	1.5503E−24	1.18666E−21
CC	GO:0031674	I band	49	4	53	76	3.73754E−24	2.69256E−21
BP	GO:0019752	Carboxylic acid metabolic process	164	42	206	610	6.64057E−24	4.51817E−21
BP	GO:0048731	System development	389	318	707	2997	4.92627E−23	3.17537E−20
BP	GO:0009653	Anatomical structure morphogenesis	250	221	471	1821	5.78295E−23	3.54119E−20

**Table 2:** Most significant enrichment of the top 20 GO term entries according to FDR value at postoperative 5 weeks

Category	GO.ID	Term	Up	Down	DEG	Total	*P* value	FDR
CC	GO:0005739	Mitochondrion	281	19	300	1111	1.93367E−68	1.88726E−64
CC	GO:0005737	Cytoplasm	689	301	990	7284	1.22918E−56	5.99839E−53
BP	GO:0055114	Oxidation–reduction process	177	29	206	838	2.26224E−38	7.35983E−35
BP	GO:0006091	Generation of precursor metabolites and energy	97	9	106	311	1.18895E−32	2.90104E−29
BP	GO:0015980	Energy derivation by oxidation of organic compounds	74	5	79	186	3.79835E−32	7.41437E−29
CC	GO:0031966	Mitochondrial membrane	106	8	114	370	4.54542E−31	7.39388E−28
BP	GO:0044281	Small molecule metabolic process	198	46	244	1230	1.74148E−29	2.42812E−26
CC	GO:0005740	Mitochondrial envelope	110	8	118	410	4.27028E−29	5.20974E−26
BP	GO:0045333	Cellular respiration	59	1	60	127	9.27649E−28	9.73815E−25
CC	GO:0005743	Mitochondrial inner membrane	82	5	87	253	9.97761E−28	9.73815E−25
MF	GO:0016491	Oxidoreductase activity	135	25	160	685	4.57839E−27	4.06228E−24
CC	GO:0019866	Organelle inner membrane	85	5	90	291	7.56808E−25	6.15537E−22
CC	GO:1990204	Oxidoreductase complex	47	2	49	104	4.06519E−23	3.05202E−20
BP	GO:0006082	Organic acid metabolic process	118	30	148	663	5.92202E−23	4.12849E−20
BP	GO:0043436	Oxoacid metabolic process	116	29	145	647	1.12659E−22	7.33036E−20
BP	GO:0030029	Actin filament-based process	67	62	129	543	1.32829E−22	8.10257E−20
CC	GO:0030016	Myofibril	44	8	52	121	3.77096E−22	2.16498E−19
CC	GO:0098798	Mitochondrial protein complex	72	2	74	231	1.20695E−21	6.54435E−19
CC	GO:0043292	Contractile fiber	45	8	53	129	1.88861E−21	9.70148E−19
CC	GO:0005759	Mitochondrial matrix	65	1	66	193	3.63184E−21	1.77234E−18

**Table 3:** Most significant enrichment of the top 20 GO term entries according to FDR value at postoperative 6 months

Category	GO.ID	Term	Up	Down	DEG	Total	*P* value	FDR
CC	GO:0005576	Extracellular region	6	120	126	1457	5.54376E−31	3.58792E−27
CC	GO:0005615	Extracellular space	5	93	98	1003	9.09151E−28	2.94201E−24
CC	GO:0031012	Extracellular matrix	2	45	47	280	4.99476E−23	1.07754E−19
BP	GO:0030198	Extracellular matrix organization	3	29	32	206	2.16642E−14	2.91876E−11
BP	GO:0007166	Cell surface receptor signaling pathway	10	106	116	1889	2.25491E−14	2.91876E−11
BP	GO:0043062	Extracellular structure organization	3	29	32	208	2.86403E−14	3.08933E−11
CC	GO:0062023	Collagen-containing extracellular matrix	1	26	27	157	5.69719E−14	4.94137E−11
BP	GO:0048731	System development	22	136	158	2997	6.10799E−14	4.94137E−11
BP	GO:0007275	Multicellular organism development	22	146	168	3275	7.24107E−14	5.17343E−11
CC	GO:0005581	Collagen trimer	1	16	17	54	7.99355E−14	5.17343E−11
BP	GO:0009653	Anatomical structure morphogenesis	15	96	111	1821	1.63518E−13	9.62078E−11
BP	GO:0009888	Tissue development	15	69	84	1244	1.53016E−12	8.25266E−10
BP	GO:0048646	Anatomical structure formation involved in morphogenesis	7	53	60	766	1.04821E−11	4.9289E−09
BP	GO:0048856	Anatomical structure development	22	154	176	3691	1.0662E−11	4.9289E−09
BP	GO:0032502	Developmental process	23	163	186	3988	1.33031E−11	5.73987E−09
BP	GO:0048513	Animal organ development	16	101	117	2138	4.21883E−11	1.70652E−08
MF	GO:0005539	Glycosaminoglycan binding	1	21	22	139	5.78714E−11	2.2032E−08
MF	GO:0044877	Protein-containing complex binding	4	50	54	730	1.05977E−10	3.64587E−08
BP	GO:0051239	Regulation of multicellular organismal process	9	100	109	1964	1.07033E−10	3.64587E−08
BP	GO:0030199	Collagen fibril organization	1	12	13	41	1.21547E−10	3.93325E−08

**Fig. 4:**
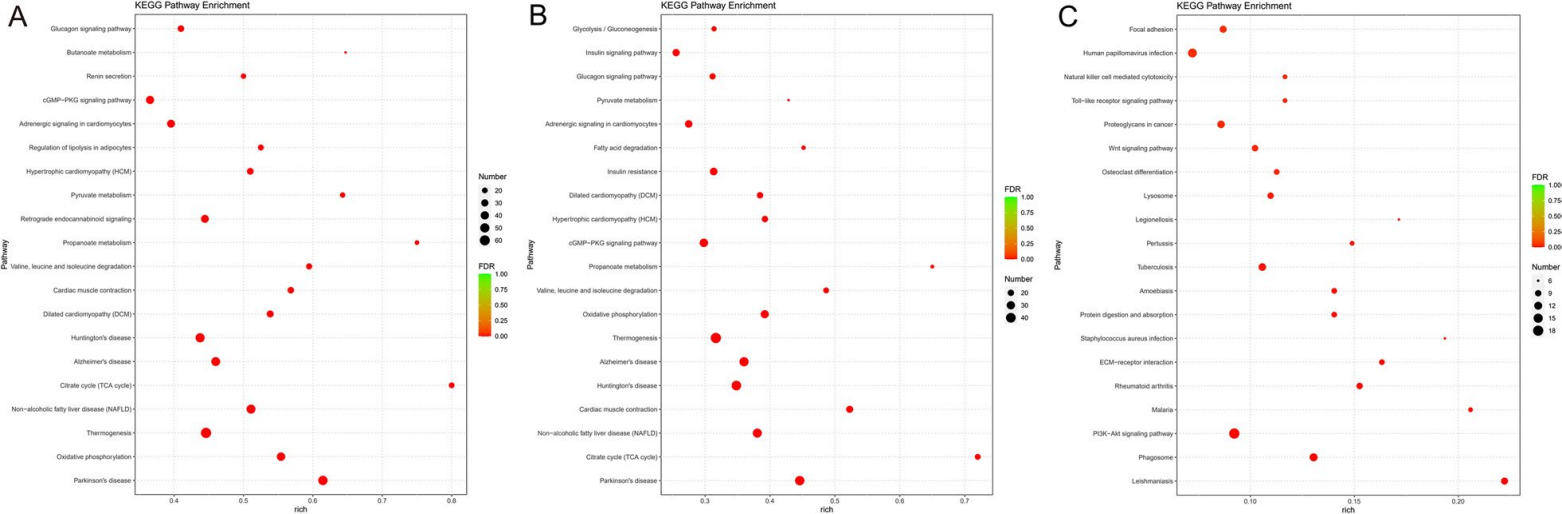
Top 20 pathways with the smallest FDR value. **A** Top 20 pathways of postoperative 1 week. **B** Top 20 pathways of postoperative 5 weeks. **C** Top 20 pathways of postoperative 6 months

As to the results of KEGG analysis, the top 20 pathways with the smallest FDR value are shown in Fig. 4. The top 3 pathways of KEGG enrichment analysis of postoperative 1 week include Parkinson's disease, oxidative phosphorylation and thermogenesis. At 5 weeks after the surgery, the top 3 pathways include Parkinson's disease, citrate cycle (TCA cycle) and non-alcoholic fatty liver disease (NAFLD). After postoperative 6 months, the top 3 pathways include leishmaniasis, phagosome and PI3K-Akt signaling pathway.

**Fig. 5:**
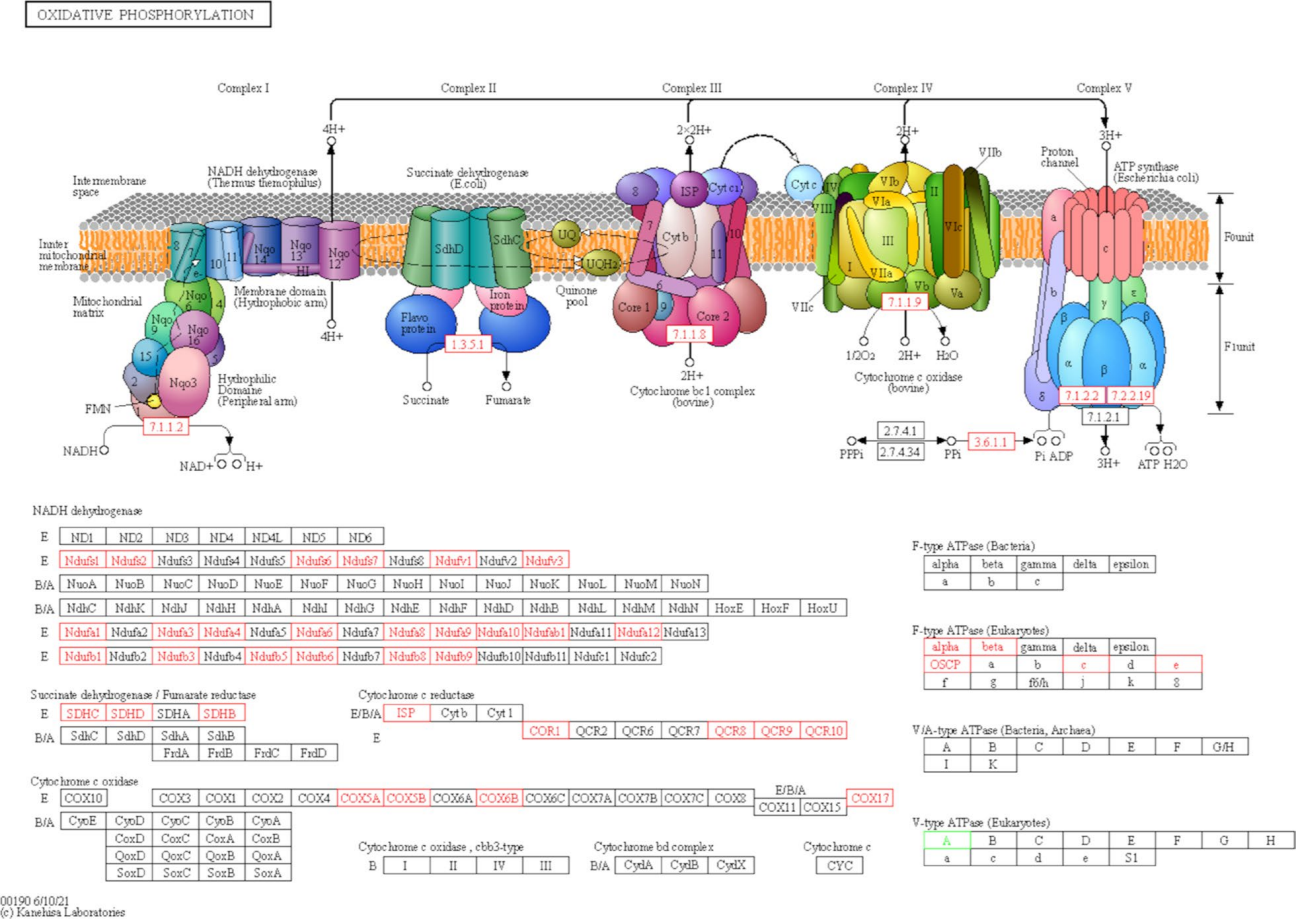
Oxidative phosphorylation pathway

The oxidative phosphorylation pathway was ranked the second top KEGG enrichment of 1 week and the eighth of 5 weeks. This pathway is shown in Fig. 5. The DEGs associated with oxidative phosphorylation at postoperative 1 week include ATP6V1A, SDHC, NDUFS1, UQCR10, ATP5MC1, NDUFB8, COX17, ATP4B, NDUFA8, NDUFAB1, NDUFA6, NDUFA1, NDUFB3, UQCRFS1, NDUFS2, ATP5PO, NDUFA9, LHPP, NDUFB9, SDHB, UQCRC1, COX5A, NDUFV1, COX5B, NDUFS6, SDHD, UQCRQ, ATP5ME, NDUFB6, NDUFB5, ATP5F1A and NDUFA10. At postoperative 5 weeks, the DEGs involved in this pathway include NDUFA8, NDUFB3, UQCRQ, NDUFA10, ATP5MC3, SDHD, COX5A, ATP5MC1, ATP5PO, NDUFS1, NDUFS2, NDUFAB1, ATP5F1A, SDHB, COX5B, UQCRFS1, NDUFB6, NDUFS6, NDUFV1, UQCRC1, NDUFB10, SDHC and NDUFA9.

The PI3K-Akt signaling pathway, which was ranked the third top KEGG enrichment of 6 months, was also ranked 91th at postoperative 1 week and 123th at postoperative 5 weeks. The PI3K-Akt signaling pathway is shown in Additional file 4: Fig. S4. The associated DEGs at postoperative 6 months include EGF, FN1, ANGPT4, SPP1, COMP, TLR4, COL1A2, CSF1R, ITGA11, CREB3L1, NGFR, COL6A1, TLR2, PCK1, THBS3, COL6A2, FGF10 and IGF1.

The osteoclast differentiation pathway, which was ranked the 14th top KEGG enrichment of 6 months, was also ranked 212th and 149th at postoperative 1 week and 5 weeks. The osteoclast differentiation pathway is shown in Additional file 5: Fig. S5. The associated DEGs at postoperative 6 months include FOS, CSF1R, NFATC2, PPARG, TYROBP, NCF2 and NCF4.

The Wnt signaling pathway, which was ranked the 15th top KEGG enrichment of 6 months, was also ranked 129th and 128th at postoperative 1 week and 5 weeks. The Wnt signaling pathway is shown in Additional file 6: Fig. S6. The associated DEGs at postoperative 6 months include WNT2B, FZD7, NFATC4, WNT16, NFATC2, SFRP2, ROR2, BAMBI and SFRP1.

**Fig. 6:**
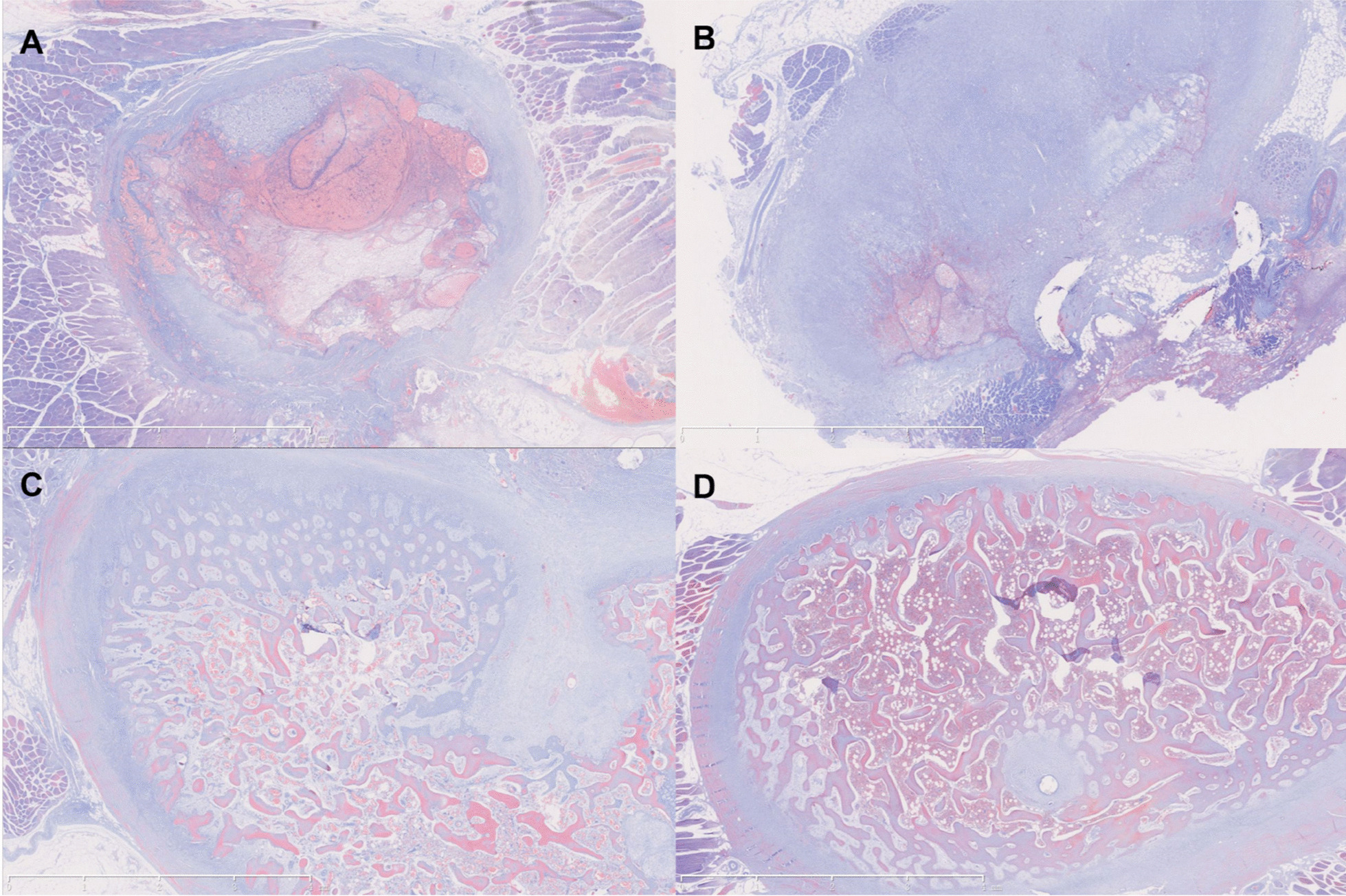
The osteogenesis conditions of periostea and newly formed bone were stained with Masson method. **A** The results show that at 3 days postoperatively, it is mainly a period of hematoma formation and a small amount of cartilage formation. **B** At 1 week postoperatively, the hematoma gradually disappeared and more cartilage was formed. **C** At 5 weeks postoperatively, the periosteal sheath was filled with cartilage tissue and new trabeculae gradually replaced the cartilage tissue. **D** At 6 months postoperatively, the periosteal sheath was already filled with new bone tissue that had been shaped

The results of periosteal and bone staining show that at postoperative 3 days, it is mainly a period of hematoma formation and a small amount of cartilage formation (Fig. 6A). At postoperative 1 week, the hematoma gradually disappeared and more cartilage was formed (Fig. 6B). At postoperative 5 weeks, the periosteal sheath was filled with cartilage tissue and new trabeculae gradually replaced the cartilage tissue (Fig. 6C). At postoperative 6 months, the periosteal sheath was already filled with new bone tissue that had been shaped (Fig. 6D).

## Discussion

Current treatments for large bone defect mainly include autologous, allogeneic and alloplastic grafts, and are very limited due to a lack of donor sites, potential immunogenic response [13, 14]. Guided self-generation has promising potential of clinical translation, since it need not exogenous cells, growth factors and scaffolds, which are necessary in traditional tissue engineered techniques [11, 12]. Periosteum is considered to be crucial in the procedure of guided self-generation, but little is known about the molecular regulation mechanisms. This study focuses on the molecular regulation mechanisms that periosteum is involved in during the bone regeneration.

The current study outlines the dynamic changes of transcription associated with periosteum in guided bone self-generation. The dynamic changes of gene expression were firstly reported in so detailed time points. The dynamic changes of the number of DEGs fluctuated very much. From the dynamically changing volatility graph of DEGs, we could find 3 volatility peaks and we chose 3 time points of DEG number peaks (1 week, 5 weeks and 6 months) to study the function of DEGs.

The cytoplasm was one of the top terms. Special chemical and physical characteristics of the extracellular matrix (ECM) could pass on the stimulus from outside the cell to the cytoplasm and then results in osteocyte cytoskeletal changes, ECM remodeling and altering bone [15]. Oxidoreductase activity was the top term both at postoperative 1 week and 5 weeks. It was reported oxidoreductase activity was inversely correlated with collagen synthesis in osteoblasts [16]. Oxidation–reduction process was also the top term. Oxidation–reduction process was significantly dysregulated following the formation of heterotopic ossification [17]. Genes in the oxidation–reduction process category were down-regulated in the healing bones between titanium orthodontic mini-implants site bone and sites of surgical defects [18]. The top terms suggest oxidoreductase reaction has an important role in bone regeneration. Mitochondrion was the top term of postoperative 5 weeks, and mitochondrion plays important role in generating reactive oxygen species (ROS), thus affecting bone remodeling [19, 20]. Extracellular region and extracellular matrix organization were the top terms of postoperative 6 months. Extracellular matrix such as enzymes and ions is often involved in bone metabolism. Extracellular Zn^2+^ and its transporters participate in regulating physiological or pathological processes [20, 21]. Nucleotide-metabolizing ectoenzymes has been reported to be associated with bone modeling [22–24]. However, the important role of these enzymes in bone metabolism has not been fully understood yet.

Oxidative phosphorylation is one of the top pathways both at postoperative 1 week and 5 weeks. It is consistent with the results of GO analysis. Studies have found there existed a metabolic shift for stem cell differentiation [25–27]. Glycolytic metabolism is the main metabolism in stem cells for their self-renewal, and it has a benefit for cell proliferation [28]. There is an increased cellular energy demand when stem cells commit to differentiate. During this procedure, glycolytic metabolism could be shifted to mitochondrial oxidative phosphorylation (OXPHOS). Ram et al. investigated mitochondrial biogenesis during differentiation of periosteum-derived mesenchymal stem cells (POMSC), which express the typical mesenchymal stem cells (MSC) surface markers and have multipotency [28, 29]. Mitochondrial proteins including OXPHOS and VDAC, and mitochondrial DNA (mtDNA) contents were all increased during the differentiation of POMSC. However, glycolytic metabolism is decreased during osteogenic differentiation. Furthermore, osteogenic differentiation of POMSC could be prevented by reducing mtDNA content with ethidium bromide treatments. Therefore, OXPHOS may play a key role in the differentiation of POMSC. POMSC differentiation and regenerative medicine could be regulated by modulation of OXPHOS with pharmaceutical [30, 31].

The PI3K-Akt signaling pathway was ranked the third top KEGG enrichment of 6 months. It was reported that FN1 could activate PI3K-Akt signaling pathway to modulate the biology of cells [32]. The up-regulation of PI3K-Akt signaling pathway has been reported to stimulate fracture healing [33, 34]. Heli et al. [35] found that overexpression of FN1 could make contributions to fracture healing through the activation of TGF-β/PI3K-Akt signaling pathway. In this current study, FN1 was one of the up-regulated DEGs. We could infer that the FN1 may activate the PI3K-Akt signaling pathway to affect bone self-generation. Concetta et al. reported that periosteum-derived progenitor cells could be induced toward osteoblastic differentiation under IGF1 or IGF1/VEGF stimuli with the activation of PI3K-Akt signaling pathway, which plays important role in osteogenesis processes [36]. The IGF1 was also differentially up-regulated in our study, which suggests that IGF1 may affect the activation of PI3K-Akt signaling pathway, thus regulating the bone regeneration.

The osteoclast differentiation pathway was ranked the 14th top KEGG enrichment of 6 months. The function coordination of skeletal cells which include bone-forming osteoblasts and bone-resorbing osteoclasts maintains the homeostasis of bone [37]. Many factors affect the differentiation of osteoclasts, including receptor activator of nuclear factor (NF)-κB ligand (RANKL), tumor necrosis factor (TNF) family cytokine and macrophage colony-stimulating factor (M-CSF) [38]. The c-Fos is an essential molecule for osteoclastogenesis and was up-regulated. We speculate that the activation of this pathway is mainly associated with callus shaping at different time points. The Wnt signaling pathway was top pathway of 3 time points. It was reported that Wnt signaling was involved in different cellular activities during cell differentiation, organogenesis and early embryonic development [39]. Fracture repair was considered to be a recapitulation of embryonic development, and members of the Wnt signaling pathway were activated [40]. Nan et al. found that Wnt signaling pathway was activated during bone regeneration [40]. The Wnt signaling pathway may play an important role in the regenerative process.

Several strategies, including gene therapy and tissue engineering together with mesenchymal stem cells (MSC), have been proposed to promote the healing of the musculoskeletal tissue. Moreover, a recent technology has revolutionized gene editing: Clustering regulatory interval short palindromic repeats (CRISPR) features simple target design, affordable, versatile and efficient, but requires more research to be the preferred platform for genome editing. Predictive genomics DNA analysis can understand which genetic advantages (if any) can be exploited and why specific rehabilitation programs are more effective in some people than others [41, 42]. Therefore, a better understanding of the genetic impact on musculoskeletal system function and disease healing is needed to plan and develop patient-specific management strategies. Currently, while some results are promising, all biological interventions are experimental and the cost/effectiveness has not been proven. In addition, the short follow-up time of most studies questioned the durability of treatment [42]. In this study, autologous periosteum was used to guide bone regeneration in vivo, and the regenerated bone was used for precise repair of the body. This technology has a promising clinical application prospect. The related differential genes and signaling pathways identified in this study can provide a rich theoretical basis for later gene and molecular intervention.

Though this in vivo study provides a better understanding in the molecular mechanisms involved in guided self-generation, it also has limitations. First, micro-CT can be conducted to evaluate the conditions of bone regeneration at different time points. The association of changes of molecules and bone regeneration can be analyzed to provide more precise explanations of the mechanisms. Besides, different parts of the regeneration may be at different stage of healing process. Immuno-histochemistry to localize the transcript and protein expression may provide deeper appreciation of the function of specific genes.

## Conclusions

This study shows the guided bone regeneration involves DEGs associated with oxidation–reduction process, mitochondrion and oxidoreductase activity. The main signaling pathways includes oxidative phosphorylation, PI3K-Akt signaling pathway, osteoclast differentiation pathway and Wnt signaling. This study could deepen our understanding of the molecular mechanisms involved in the guided bone regeneration. With these findings of molecular changes at different time points, it gains the potential of regulating the specific mechanism to enhance the bone regeneration.

## Supplementary Information

**Additional file 1: Figure S1**. Volcano maps of the DEGs between control and trauma group at postoperative different time points (A: 1 day, B: 3 days, C: 1 week, D: 2 weeks, E: 1 month, F: 5 weeks, G: 3 months, H: 6 months, I: 7 months).

**Additional file 2: Figure S2**. Results of cluster analysis revealed unknown biological connections between genes through expression clustering.

**Additional file 3: Figure S3**. Top 10 GO term entries with the smallest p-value. A, The top 10 GO term entries of postoperative 1 week. B, The top 10 GO term entries of postoperative 5 weeks. C, The top 10 GO term entries of postoperative 6 months.

**Additional file 4: Figure S4**. PI3K-Akt signaling pathway.

**Additional file 5: Figure S5**. Osteoclast differentiation pathway.

**Additional file 6: Figure S6**. Wnt signaling pathway.

## Data Availability

The datasets used and/or analyzed during the current study are available from the corresponding author on reasonable request.

## References

[1] Fan W, Crawford R, Xiao Y (2008). Structural and cellular differences between metaphyseal and diaphyseal periosteum in different aged rats. Bone.

[2] Matsushima S, Isogai N, Jacquet R, Lowder E, Tokui T, Landis WJ (2011). The nature and role of periosteum in bone and cartilage regeneration. Cells Tissues Organs.

[3] Lin Z, Fateh A, Salem DM, Intini G (2014). Periosteum: biology and applications in craniofacial bone regeneration. J Dent Res.

[4] Augustin G, Antabak A, Davila S (2007). The periosteum. Part 1: anatomy, histology and molecular biology. Injury.

[5] Colnot C, Zhang X, Knothe Tate ML (2012). Current insights on the regenerative potential of the periosteum: molecular, cellular, and endogenous engineering approaches. J Orthop Res.

[6] Colnot C (2009). Skeletal cell fate decisions within periosteum and bone marrow during bone regeneration. J Bone Miner Res.

[7] Mesgarzadeh AH, Abadi A, Keshani F (2019). Seven-year follow-up of spontaneous bone regeneration following segmental mandibulectomy: alternative option for mandibular reconstruction. Dent Res J.

[8] Sharma P, Williams R, Monaghan A (2013). Spontaneous mandibular regeneration: another option for mandibular reconstruction in children. Br J Oral Maxillofac Surg.

[9] Ahmad O, Omami G (2015). Self-regeneration of the mandible following hemimandibulectomy for ameloblastoma: a case report and review of literature. J Maxillofac Oral Surg.

[10] Wei J, Herrler T, Han D (2016). Autologous temporomandibular joint reconstruction independent of exogenous additives: a proof-of-concept study for guided self-generation. Sci Rep.

[11] Wei J, Herrler T, Dai C, Liu K, Han D, Li Q (2016). Guided self-generation of vascularized neo-bone for autologous reconstruction of large mandibular defects. J Craniofac Surg.

[12] Wei J, Herrler T, Liu K (2016). The role of cell seeding, bioscaffolds, and the in vivo microenvironment in the guided generation of osteochondral composite tissue. Tissue Eng Part A.

[13] Warnke PH, Springer IN, Wiltfang J (2004). Growth and transplantation of a custom vascularised bone graft in a man. Lancet Lond Engl.

[14] Xu H, Han D, Dong JS (2010). Rapid prototyped PGA/PLA scaffolds in the reconstruction of mandibular condyle bone defects. Int J Med Robot Comput Assist Surg MRCAS.

[15] Camal Ruggieri IN, Cícero AM, Issa J, Feldman S (2021). Bone fracture healing: perspectives according to molecular basis. J Bone Miner Metab.

[16] Zhang X, Zhao G, Zhang Y (2018). Activation of JNK signaling in osteoblasts is inversely correlated with collagen synthesis in age-related osteoporosis. Biochem Biophys Res Commun.

[17] Xie K, Chen L, Yang J (2021). RNA sequencing evidences the prevention of oxidative stress is effective in injury-induced heterotopic ossification treatment. J Biomed Nanotechnol.

[18] Nahm KY, Heo JS, Lee JH (2015). Gene profiling of bone around orthodontic mini-implants by RNA-sequencing analysis. Biomed Res Int.

[19] Vacek TP, Kalani A, Voor MJ, Tyagi SC, Tyagi N (2013). The role of homocysteine in bone remodeling. Clin Chem Lab Med.

[20] Levaot N, Hershfinkel M (2018). How cellular Zn(2+) signaling drives physiological functions. Cell Calcium.

[21] Malavasi F, Deaglio S, Zaccarello G (2010). The hidden life of NAD+-consuming ectoenzymes in the endocrine system. J Mol Endocrinol.

[22] Faulkner G, Lanfranchi G, Valle G (2001). Telethonin and other new proteins of the Z-disc of skeletal muscle. IUBMB Life.

[23] Goley ED, Rammohan A, Znameroski EA, Firat-Karalar EN, Sept D, Welch MD (2010). An actin-filament-binding interface on the Arp2/3 complex is critical for nucleation and branch stability. Proc Natl Acad Sci USA.

[24] Lobo JG, Leite AL, Pereira HA (2015). Low-level fluoride exposure increases insulin sensitivity in experimental diabetes. J Dent Res.

[25] Chen CT, Shih YR, Kuo TK, Lee OK, Wei YH (2008). Coordinated changes of mitochondrial biogenesis and antioxidant enzymes during osteogenic differentiation of human mesenchymal stem cells. Stem Cells (Dayton, Ohio).

[26] Varum S, Rodrigues AS, Moura MB (2011). Energy metabolism in human pluripotent stem cells and their differentiated counterparts. PLoS ONE.

[27] Hofmann AD, Beyer M, Krause-Buchholz U, Wobus M, Bornhäuser M, Rödel G (2012). OXPHOS supercomplexes as a hallmark of the mitochondrial phenotype of adipogenic differentiated human MSCs. PLOS ONE.

[28] Lee AR, Moon DK, Siregar A (2019). Involvement of mitochondrial biogenesis during the differentiation of human periosteum-derived mesenchymal stem cells into adipocytes, chondrocytes and osteocytes. Arch Pharmacal Res.

[29] Brunt KR, Weisel RD, Li RK (2012). Stem cells and regenerative medicine—future perspectives. Can J Physiol Pharmacol.

[30] Mara CS, Sartori AR, Duarte AS, Andrade AL, Pedro MA, Coimbra IB (2011). Periosteum as a source of mesenchymal stem cells: the effects of TGF-β3 on chondrogenesis. Clinics (Sao Paulo, Brazil).

[31] Murphy MP, Hartley RC (2018). Mitochondria as a therapeutic target for common pathologies. Nat Rev Drug Discov.

[32] Xiang L, Xie G, Ou J, Wei X, Pan F, Liang H (2012). The extra domain A of fibronectin increases VEGF-C expression in colorectal carcinoma involving the PI3K/AKT signaling pathway. PLOS ONE.

[33] Kita K, Kimura T, Nakamura N, Yoshikawa H, Nakano T (2008). PI3K/Akt signaling as a key regulatory pathway for chondrocyte terminal differentiation. Genes Cells Devot Mol Cell Mech.

[34] Li G, Wang L, Jiang Y (2017). Upregulation of Akt signaling enhances femoral fracture healing by accelerating atrophic quadriceps recovery. Biochim Biophys Acta Mol Basis Dis.

[35] Zhang H, Chen X, Xue P, Ma X, Li J, Zhang J (2021). FN1 promotes chondrocyte differentiation and collagen production via TGF-β/PI3K/Akt pathway in mice with femoral fracture. Gene.

[36] Ferretti C, Vozzi G, Falconi M (2014). Role of IGF1 and IGF1/VEGF on human mesenchymal stromal cells in bone healing: two sources and two fates. Tissue Eng Part A.

[37] Sato K, Suematsu A, Nakashima T (2006). Regulation of osteoclast differentiation and function by the CaMK-CREB pathway. Nat Med.

[38] Asagiri M, Takayanagi H (2007). The molecular understanding of osteoclast differentiation. Bone.

[39] Moon RT, Bowerman B, Boutros M, Perrimon N (2002). The promise and perils of Wnt signaling through beta-catenin. Science (New York, NY).

[40] Zhong N, Gersch RP, Hadjiargyrou M (2006). Wnt signaling activation during bone regeneration and the role of Dishevelled in chondrocyte proliferation and differentiation. Bone.

[41] Aicale R, Tarantino D, Maccauro G, Peretti GM, Maffulli N (2019). Genetics in orthopaedic practice. J Biol Regul Homeost Agents.

[42] Andia I, Maffulli N (2019). New biotechnologies for musculoskeletal injuries. Surgeon.

