# Supplementary figures and images for: Continuous dynamic identification of key genes and molecular signaling pathways of periosteum in guided bone self-generation in swine model
Source: J Orthop Surg Res. 2023 Jan 18;18:53. doi: 10.1186/s13018-023-03524-y (PMC9847205; doi:10.1186/s13018-023-03524-y)

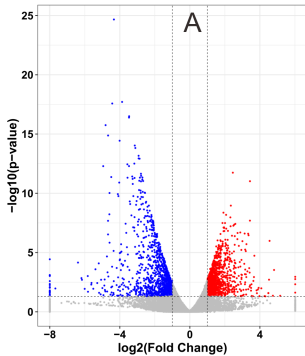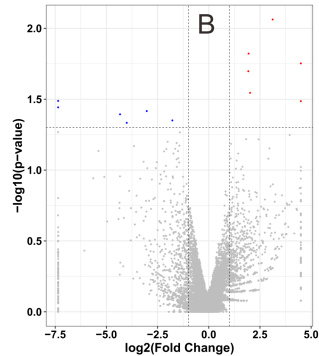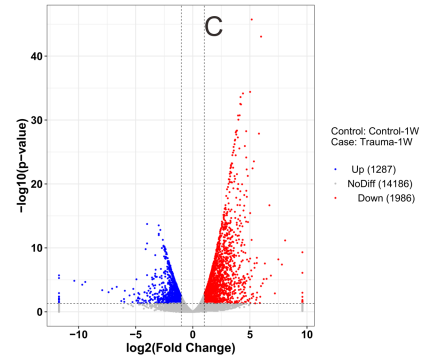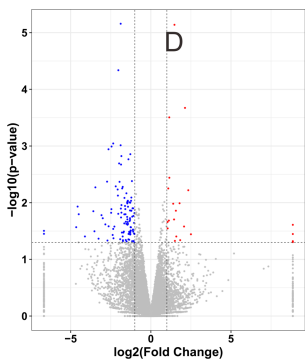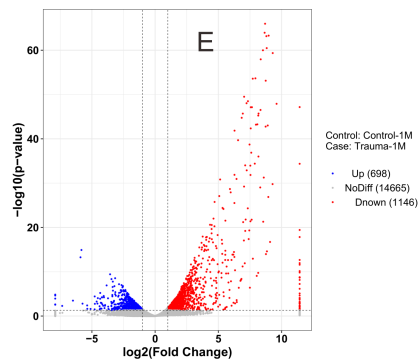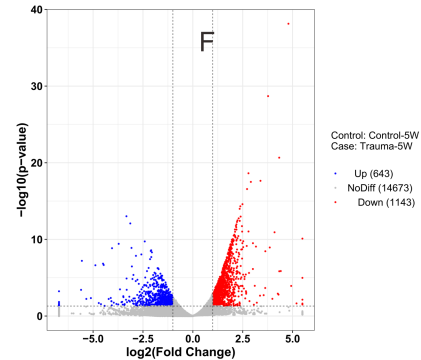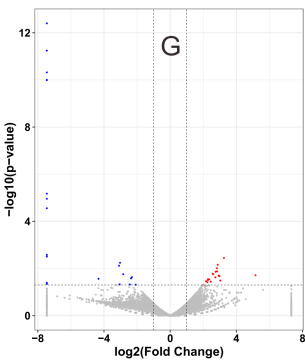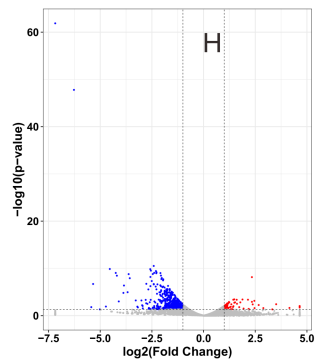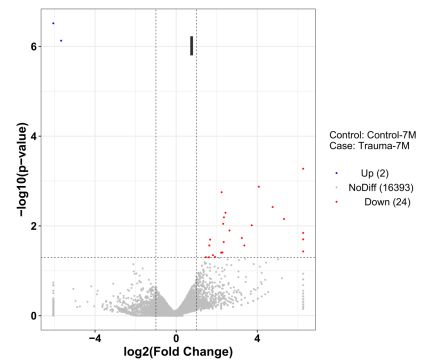

Supplement: Supplementary file 1 — Additional file 1: Figure S1. Volcano maps of the DEGs between control and trauma group at postoperative different time points (A: 1 day, B: 3 days, C: 1 week, D: 2 weeks, E: 1 month, F: 5 weeks, G: 3 months, H: 6 months, I: 7 months). [file 13018_2023_3524_MOESM1_ESM.pdf]

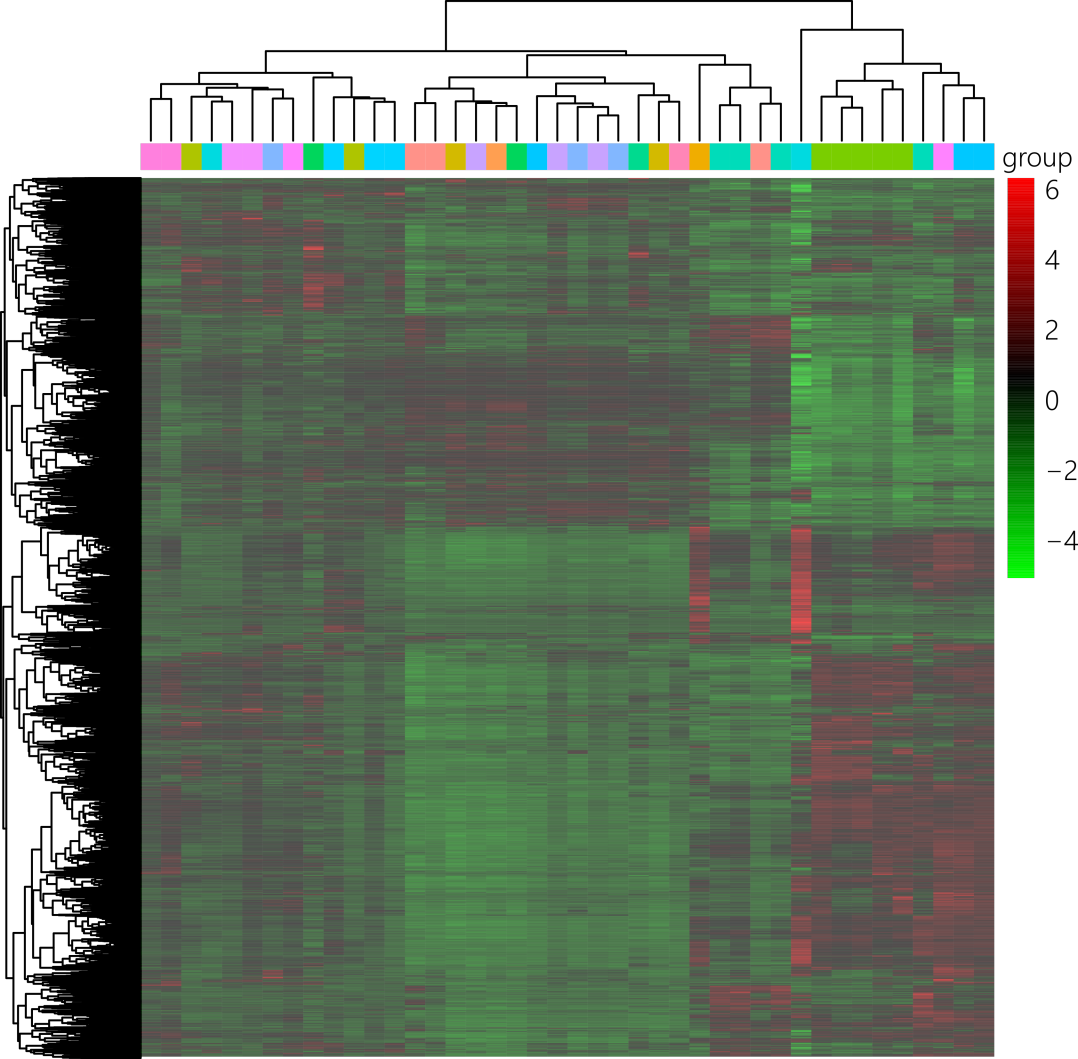

Supplement: Supplementary file 2 — Additional file 2: Figure S2. Results of cluster analysis revealed unknown biological connections between genes through expression clustering. [file 13018_2023_3524_MOESM2_ESM.pdf]

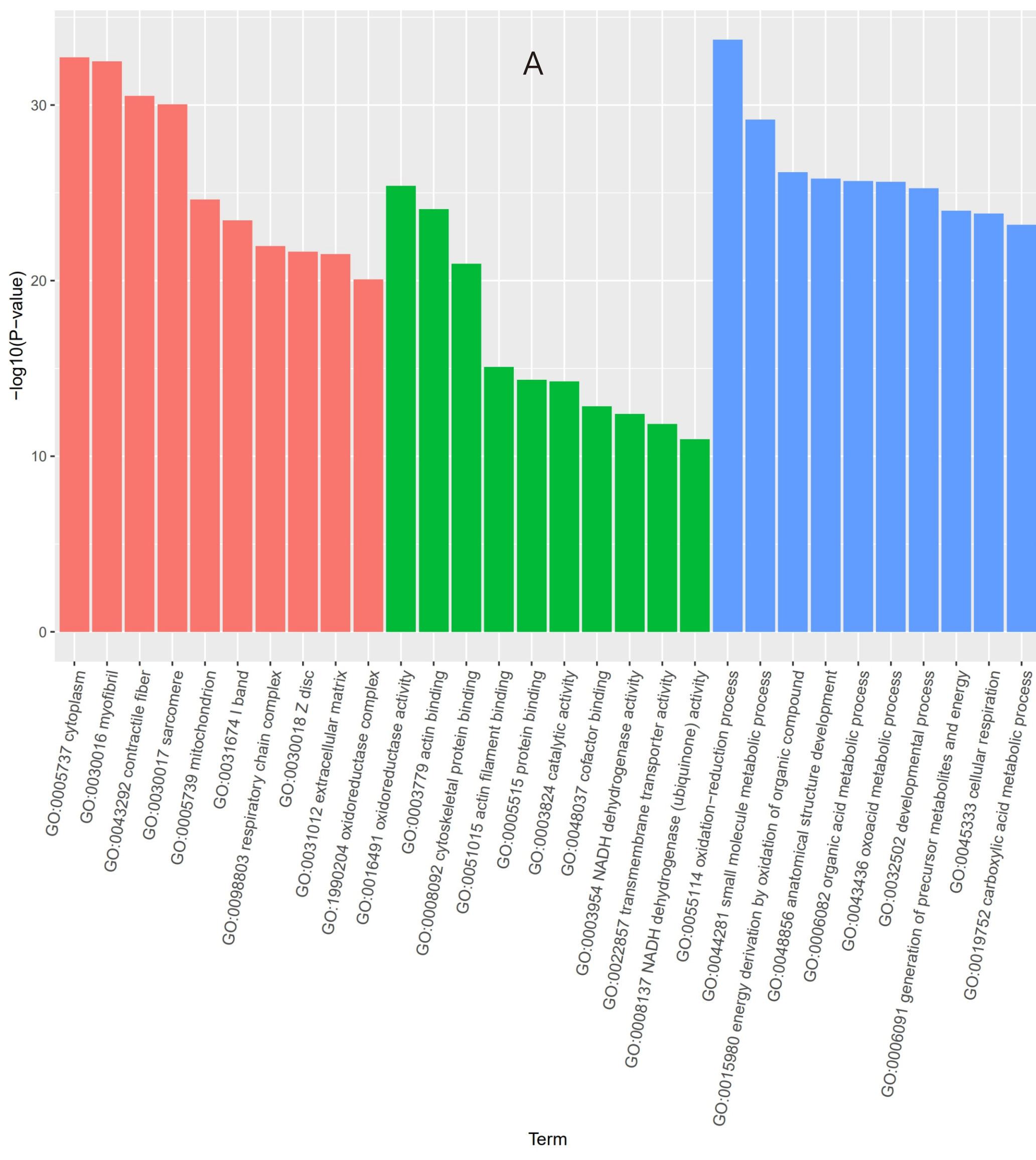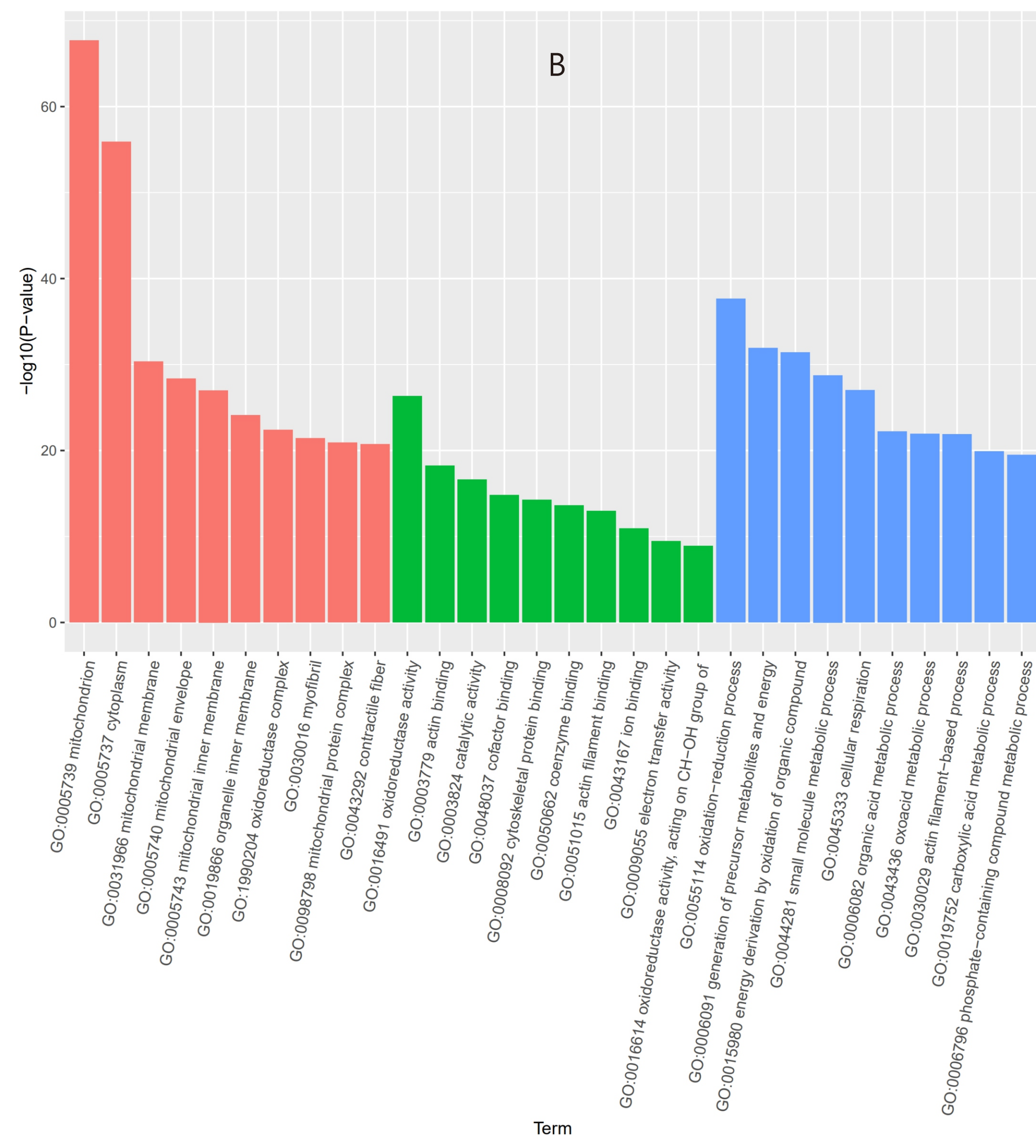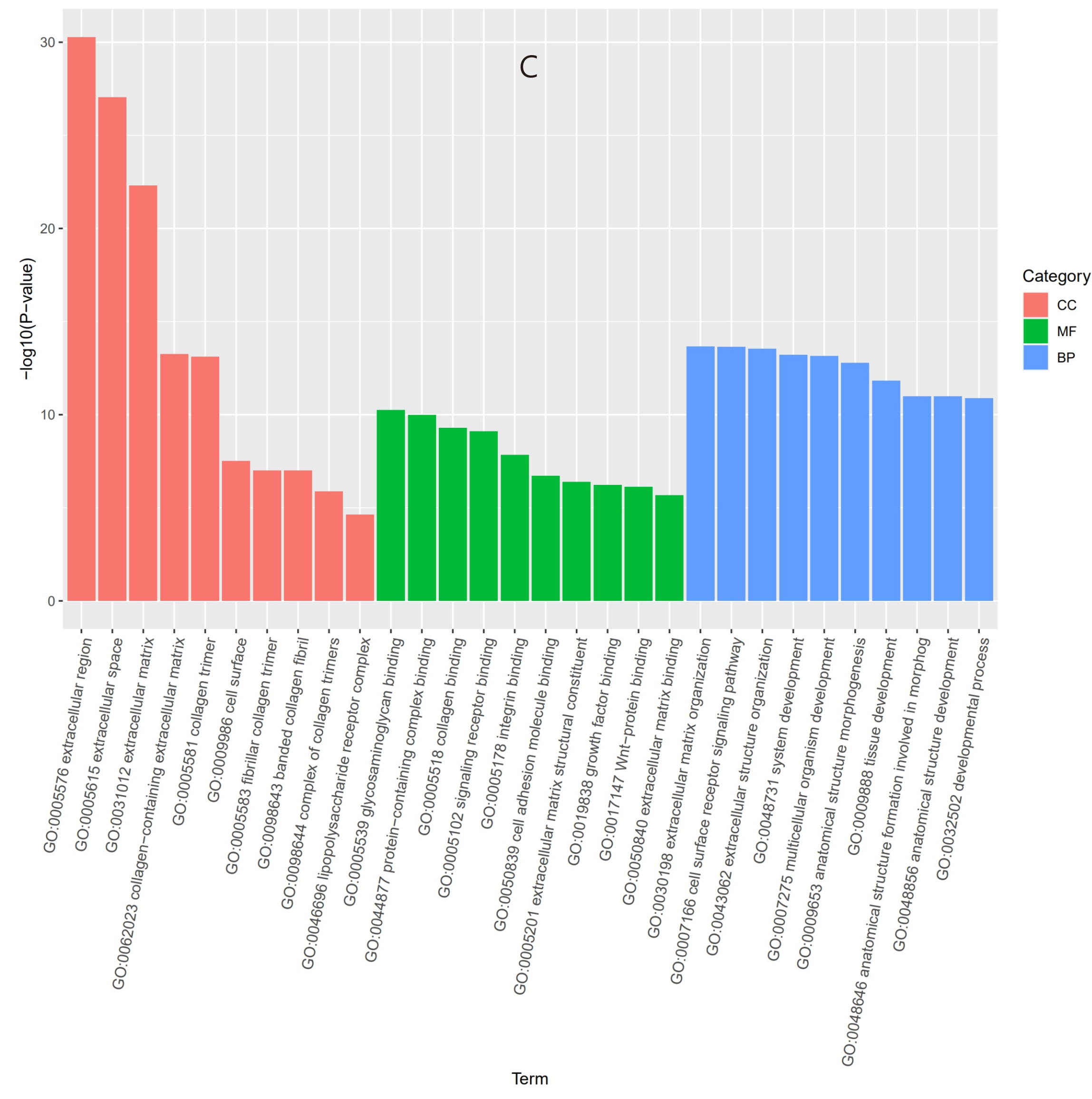

Supplement: Supplementary file 3 — Additional file 3: Figure S3. Top 10 GO term entries with the smallest p-value. A, The top 10 GO term entries of postoperative 1 week. B, The top 10 GO term entries of postoperative 5 weeks. C, The top 10 GO term entries of postoperative 6 months. [file 13018_2023_3524_MOESM3_ESM.pdf]

# PI3K-AKT SIGNALING PATHWAY

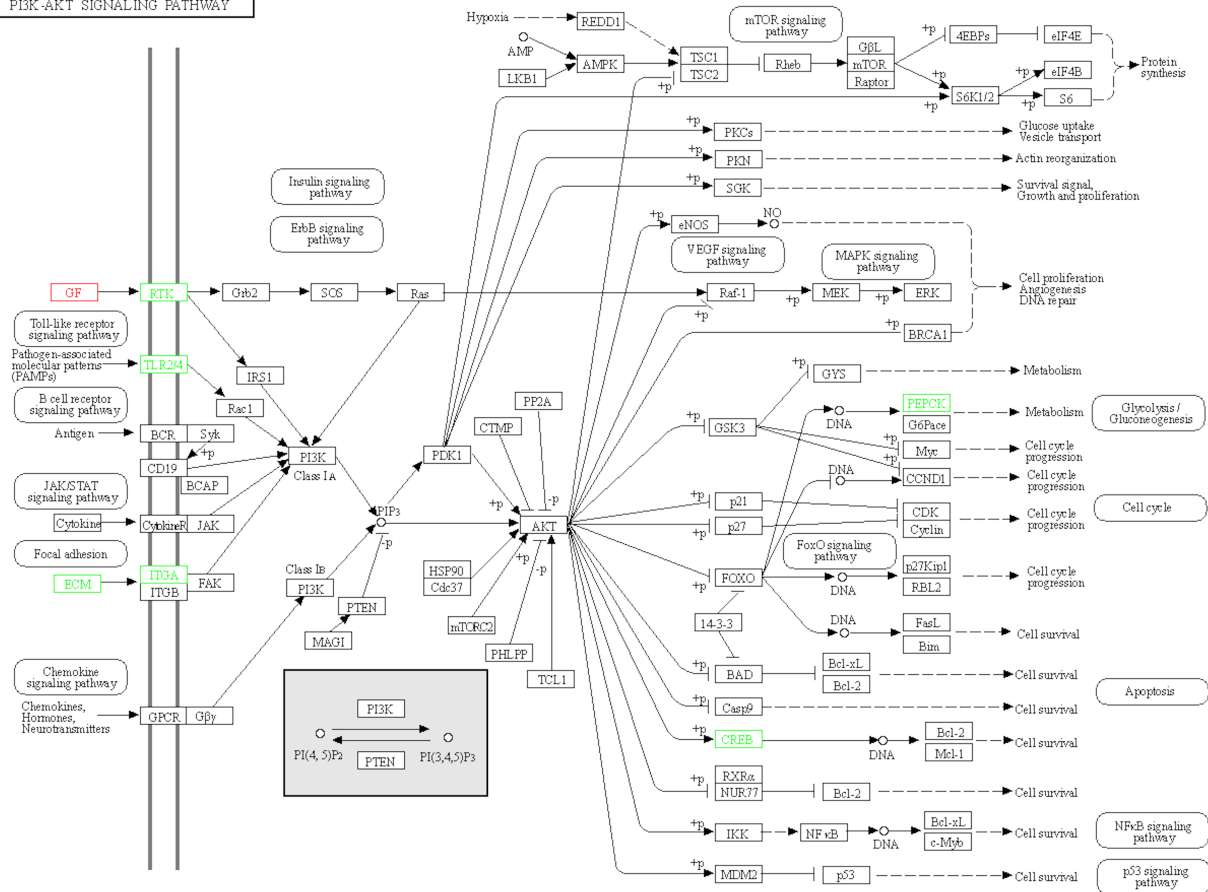

Supplement: Supplementary file 4 — Additional file 4: Figure S4. PI3K-Akt signaling pathway. [file 13018_2023_3524_MOESM4_ESM.pdf]

# OSTEOCLAST DIFFERENTIATION

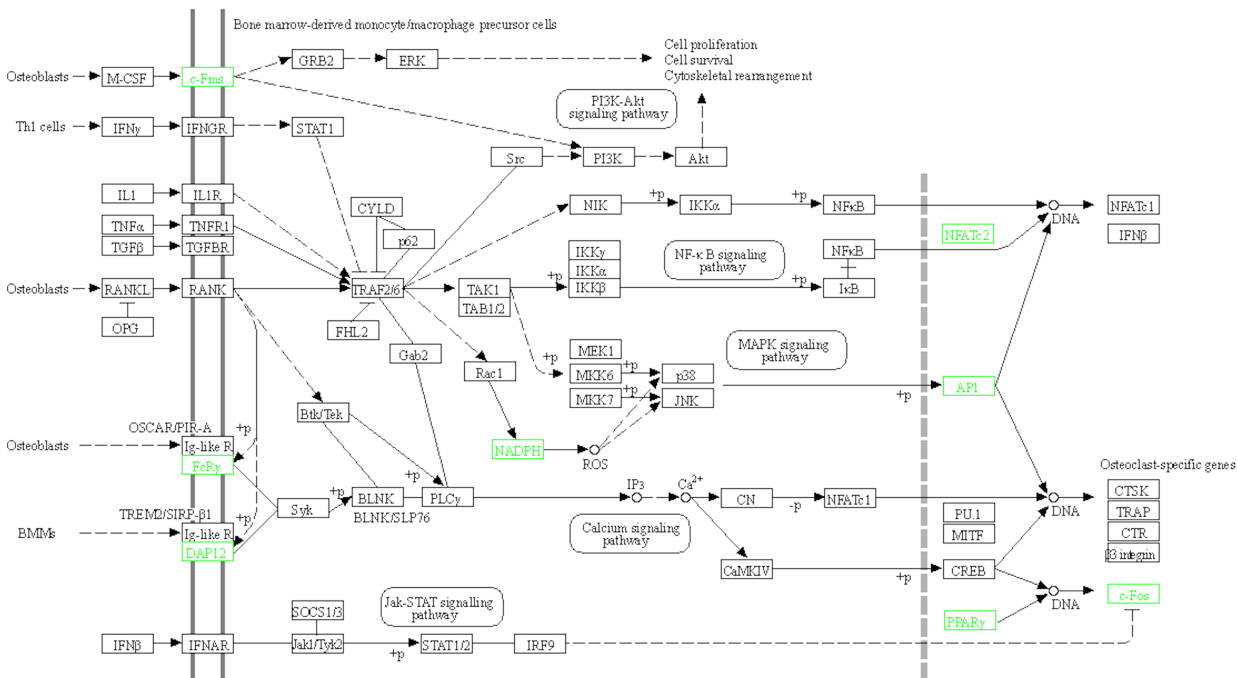

Supplement: Supplementary file 5 — Additional file 5: Figure S5. Osteoclast differentiation pathway. [file 13018_2023_3524_MOESM5_ESM.pdf]

# WNT SIGNALING PATHWAY

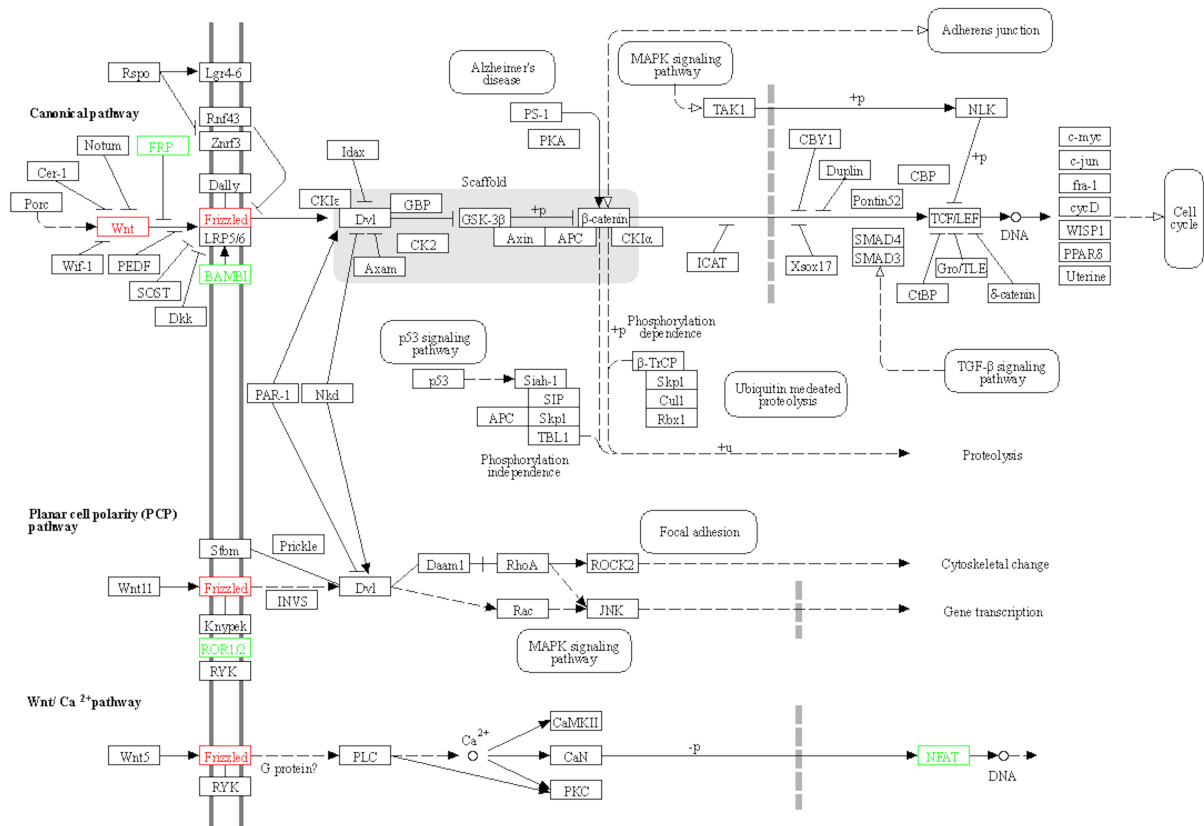

Supplement: Supplementary file 6 — Additional file 6: Figure S6. Wnt signaling pathway. [file 13018_2023_3524_MOESM6_ESM.pdf]
